# Validation of sample pooling for high-throughput RT-qPCR subtyping of avian influenza A(H5)

**DOI:** 10.1128/spectrum.00850-26

**Published:** 2026-07-13

**Authors:** Grant P. Higerd-Rusli, Seth A. Hoffman, Abraar Karan, ChunHong Huang, Malaya K. Sahoo, Ingrid E. A. Morante, Matthew M. Hernandez, Benjamin A. Pinsky

**Affiliations:** 1 Department of Pathology, Stanford University School of Medicine 10624, Stanford, California, USA; 2 Division of Infectious Diseases & Geographic Medicine, Department of Medicine, Stanford University School of Medicine 10624, Stanford, California, USA; 3 Clinical Virology Laboratory, Stanford Health Care, Stanford, California, USA; Montefiore Medical Center and Albert Einstein College of Medicine, Bronx, New York, USA

**Keywords:** avian influenza, influenza, H5, HPAI, PCR, pooled testing

## Abstract

Highly pathogenic avian influenza A(H5N1) is a global health threat due to its high historical virulence and increasing mammalian host range, as demonstrated by the 2024–2025 outbreak among U.S. dairy cattle, with 71 confirmed human infections. While human-to-human transmission has not yet been documented, limited pre-existing human immunity, and uncertainty about evolutionary barriers to human infection make H5N1 a potential pandemic threat. Expanding testing capacity for H5N1 beyond its currently limited scope would be critical for response to potential outbreaks. Thus, we developed and validated a dual-target reverse transcription PCR assay for H5 subtyping of pooled influenza A-positive samples. The assay targets two regions of the hemagglutinin (HA) gene of influenza A(H5). Using contrived samples containing inactivated bovine H5N1 virus, we determined the assay’s 95% lower limit of detection for individual samples (58 copies/mL; 95% confidence interval [CI]: 50 to 66 copies/mL) and eight-sample pools (2,465 copies/mL; 95% CI: 1,969 to 3,756 copies/mL). The assay demonstrated 100% positive percent agreement, detecting H5 in all contrived H5-positive pools (32/32 pools; 95% CI: 89.1% to 100.0%) where the contrived positive samples ranged from 500,000 to 3,906 copies/mL. We observed 100% negative percent agreement in H5-negative, influenza A-positive pools (32/32; 95% CI: 89.1% to 100.0%). Though sample pooling reduced analytical sensitivity, contrived samples with H5 virus present at levels typically found in human respiratory infections were detected in eight-sample pools. This approach has the potential to increase testing capacity for influenza A (H5) while maintaining adequate sensitivity, enhancing preparedness for potential outbreaks.

**IMPORTANCE:**

Avian influenza A(H5N1) poses a potential pandemic threat. H5N1 testing capacity is currently limited. We validated an influenza A (H5) subtyping RT-qPCR for testing pooled samples to increase capacity. The assay displays adequate analytical sensitivity to detect clinical respiratory infections based on prior human cases. This proactive assay validation enhances outbreak preparedness.

## INTRODUCTION

Since its first outbreak in humans in 1996, highly pathogenic avian influenza A(H5N1) has caused widespread outbreaks among wild and domestic bird populations, as well as sporadic human infections with severe disease (1, 2). H5N1 clade 2.3.4.4b emerged in 2020 and has demonstrated its ability to infect an increasingly broad range of mammalian species (3). In 2024–2025, there was a widespread outbreak in U.S. dairy cattle caused by clade H5N1 2.3.4.4b genotype B3.13 that was associated with 71 confirmed human infections—the majority of which had known animal exposures, but several lacked clearly identifiable risk factors (4–6). Most human infections with genotype B3.13 have caused mild illness. In contrast, there have been several recent severe or fatal cases due to H5N1 genotype D1.1 and H5N5 viruses, which also belong to H5 clade 2.3.4.4b (7–12). While human-to-human transmission has not yet been documented, uncertainty remains regarding the barriers preventing such transmissibility (13). Serological evidence indicates limited pre-existing immunity in the human population, suggesting that rapid dissemination may be possible if the virus develops human transmissibility (14, 15).

Testing capacity is critical for effective outbreak response, especially for pathogens with asymptomatic transmission. However, early in an outbreak of a novel pathogen, limited capacity often necessitates restricting testing to individuals with specific exposure histories—as occurred during the early COVID-19 pandemic—potentially allowing community circulation to go undetected (16). Similarly, human testing for H5N1 is currently limited to symptomatic individuals with documented exposure risks, though testing may be offered to asymptomatic individuals with high-risk exposures (17, 18). Overcoming limited testing capacity could enable identification and isolation of infected individuals to prevent an outbreak from becoming an epidemic or pandemic. For H5N1, increased H5 subtyping capacity would be particularly important should an outbreak occur during the respiratory virus season, with co-circulation of seasonal influenza A.

Pooled or group testing of clinical samples is an efficient method to enhance testing throughput during outbreaks, increasing diagnostic capacity while managing resource constraints (16, 19–21). In a pooled testing strategy, if a pool tests positive, then the samples within the pool are tested individually (i.e., deconvoluted) to identify cases. Pooled testing is most effective at reducing cost and increasing capacity when applied to diseases with low prevalence, so that few pools need to be deconvoluted (22).

## MATERIALS AND METHODS

We developed and validated a dual-target H5 subtyping reverse transcription quantitative PCR (RT-qPCR) assay for testing pools of eight samples already known to be positive for influenza A. This assay is adapted from a previously described assay designed for detection of H5 in individual samples (23). The primer/probe sets were optimized to detect two distinct regions of the H5 clade 2.3.4.4b hemagglutinin (HA) gene and are unchanged from the previous publication (Table 1). These H5 primer/probe sets were combined in multiplex with primers and probes for RNase *P* as internal control. We removed the pan-influenza A matrix primers and probe, which were present in the previously published assay, as the test described here is intended to be used on pools of samples already known to be positive for influenza A. This removal prevents the matrix gene target, which would be present in all eight samples of a pool, from outcompeting the H5 gene target, which is likely to be present in only one (if any) of the pooled samples, and at much lower levels.

**TABLE 1:** Influenza A (H5) multiplex RT-qPCR primers and probes^*a*^^,^^*b*^

Target	Name	Sequence (5′−3′)	Final concentration
A (H5)	FluA_H5_v4_1F	TACCAGATACTGTCAATTTATTCAAC	400 nM
	FluA_H5_v4_1R	GTAACGACCCATTGGAGCACATCC	400 nM
	FluA_H5_v4_1Prb.FAM	FAM-CTGGCAATC/ZEN/ATG**R**T**R**GCTGGTCT-3IABkFQ	200 nM
	FluA_H5_v4_2F	TGGGTACCATCATAGCAATGAGCA	400 nM
	FluA_H5_v4_2R	AACTCCCTTCCAACTGCCTCAAA	400 nM
	FluA_H5_v4_2Prb.FAM	FAM-TGGGTACGC/ZEN/TGCGGACAAAGAATCCA-3IABkFQ	200 nM
Human RNase *P*	RNase *P* Fwd	AGATTTGGACCTGCGAGCG	100 nM
	RNase *P* Rev	GAGCGGCTGTCTCCACAAGT	100 nM
	RNase *P* Probe CF560	CF560-TTCTGACCTGAAGGCTCTGCGCG-BHQ-1	50 nM

^*a*^ BHQ, Black Hole Quencher; CF560, CalFluor 560; FAM, 6-carboxyfluorescein; M, matrix gene; H5 probes were purchased from Integrated DNA Technologies (IDT). The probes contain the internal quencher, ZEN, in addition to the 3′ Iowa Black quencher FQ (3IABkFQ) (proprietary to IDT). Human RNase P probes were purchased from Biosearch Technologies.

^*b*^ Sequences covered by mixed bases are in bold.

## RESULTS

We evaluated the performance of the assay using inactivated H5N1 clade 2.3.4.4b virus obtained through BEI Resources (NIAID, NIH: influenza A virus, A/bovine/Ohio/B24OSU-439/2024 [H5N1], Catalog No. NR-59886). This is a preparation of H5N1 virus cultured in Madin-Darby canine kidney (MDCK) cells that has been inactivated by gamma-irradiation and sonication. A standard curve based on known concentrations of single-stranded DNA (ssDNA) encoding the assay’s H5 2.3.4.4b targets (Table 2) was used to quantify the inactivated virus in copies/mL.

**TABLE 2:** Influenza A (H5) clade 2.3.4.4b single-stranded DNA sequences^*a*^

Name	H5 target sequence (5′−3′)	GenBank reference	Location
ssD_FluA_H5_T1C2	AGTAGGAACTTACCAGATACTGTCAATTTATTCAACAGCGGCAAGTTCCCTAGCACTGGCAATCATGATGGCTGGTCTATCTTTATGGATGTGCTCCAATGGGTCGTTACAGTGCAGAAT	OP499866	1,591–1,710
ssD_FluA_H5_T2C2	ATGGTTGGTATGGGTACCATCATAGCAATGAGCAGGGGAGTGGGTACGCTGCGGACAAAGAATCCACCCAAAAGGCAATAGATGGAGTTACCAATAAGGTCAACTCAATCATTGACAAAATGAACACTCAATTTGAGGCAGTTGGAAGGGAGTTTAATAACTTA	OP499866	1,107–1,270

^*a*^ Primer and probe targets are underlined. Single-stranded DNA was synthesized by Integrated DNA Technologies.

To determine the 95% lower limit of detection of the assay with individual samples, we diluted inactivated virus to 200, 100, and 50 copies/mL in viral transport media from pooled clinical nasopharyngeal swab samples negative for influenza A, influenza B, respiratory syncytial virus, adenovirus, human metapneumovirus, rhinovirus, parainfluenza virus 1–4, and SARS-CoV-2 on the Hologic Panther Fusion. Twenty replicates at each level were extracted on the QIAsymphony SP from a volume of 400 µL using the QIAsymphony DSP Virus/Pathogen Midi kit with an elution volume of 60 µL. RT-qPCR was performed using Superscript III Platinum One-Step qRT-PCR kit (Invitrogen) on the Rotor-Gene Q real-time PCR instrument (Qiagen). Each 25-µL reaction contained 12.5 µL of 2× buffer, 0.5 µL of enzyme mix, 2 µL of primer/probe mix, and 10 µL of nucleic acids. Cycling conditions were as follows: hold at 52°C for 15 min, 94°C for 2 min, then 45 cycles of 94°C for 15 s, 55°C for 40 s, and 68°C for 20 s. The fluorescence signal was analyzed in the green (H5) and yellow (RNase *P*) channels. Thresholds were set at 0.1 normalized fluorescence units for all targets. Any exponential amplification curve crossing the threshold in the green channel was interpreted as subtype H5. The 95% lower limit of detection (LLOD) of the assay for individual H5 samples was calculated using probit analysis and was 58 copies/mL (95% confidence interval [CI] = 50 to 66 copies/mL, Table 3) (24).

**TABLE 3:** 95% lower limit of detection of inactivated H5N1 virus without sample pooling^*a*^

Copies/mL VTM	Number of replicates	Number detected
200	20	20
100	20	20
50	20	16

^*a*^ 95% LLOD (individual samples) = 58 copies/mL, 95% CI = 50 to 66 copies/mL. CI, confidence interval; LLoD, lower limit of detection; VTM, viral transport media.

To determine the 95% lower limit of detection for an H5-positive sample tested within a pool of eight influenza A-positive samples, we first prepared contrived positive samples by diluting the inactivated virus to 4,000, 2,000, 1,000, and 500 copies/mL in a matrix of pooled, respiratory virus-negative clinical nasopharyngeal swab samples. To simulate a screening pool, 200 µL of each contrived sample was then combined with 1,400 µL of a separate matrix composed of pooled, influenza A-positive, H5-negative clinical nasopharyngeal swab samples collected during the 2023–2024 respiratory virus season, creating a 1:8 dilution of each H5-positive sample. The pooled samples were rocked for 15 min to ensure adequate mixing before extraction. Twenty replicates at each level were extracted and amplified as described above. The 95% LLOD for an H5-positive sample within an eight-sample pool was calculated using probit analysis and was 2,465 copies/mL (95% CI = 1,969 to 3,756 copies/mL, Table 4).

**TABLE 4:** 95% lower limit of detection of inactivated H5N1 virus with eight-sample pooling^*a*^

copies/mL VTM	Number of replicates	Number detected
4,000	20	20
2,000	20	17
1,000	20	9
500	20	4

^*a*^ 95% LLoD (pooled samples) = 2,465 copies/mL, 95% CI = 1,969 to 3,756 copies/mL. CI, confidence interval; LLoD, lower limit of detection; VTM, viral transport media.

To evaluate the positive percent agreement of the assay compared with expected results, we prepared an eight-level, twofold serial dilution of the inactivated H5 virus. This created a set of contrived positive samples ranging from 500,000 copies/mL (5.70 log_10_ copies/mL) to 3,906 copies/mL (3.59 log_10_ copies/mL). For each of these eight levels, we assembled four eight-sample pools. Each set of four eight-sample pools consisted of one contrived H5-positive sample and an equal volume from each of seven unique influenza A-positive, H5-negative clinical samples. This resulted in a total of 32 pools which underwent extraction and amplification. The H5 target was detected in 32 of 32 pools, indicating a positive percent agreement of 100% (95% CI = 89.1%–100.0%).

To evaluate the negative percent agreement of the assay, we retrospectively screened 128 unique influenza-A positive upper respiratory samples collected between 8 August 2024 and 19 November 2024. The 128 samples were organized into two 8 × 8 grids. For each grid, 16 pools were generated. This included eight “row” pools, each containing equal volumes from the eight samples in the row, and eight “column” pools, each containing equal volumes from the eight samples in the column. This resulted in a total of 32 distinct eight-sample pools. The H5 target was not detected in any of the 32 pools, indicating a negative percent agreement of 100% (95% CI = 89.1%–100.0%). These samples were previously tested individually using the H5 subtyping RT-qPCR containing the pan-influenza A matrix target, as previously described (23). The median matrix C_T_ value was 21.5 (interquartile range [IQR] = 18.5 to 27.1], confirming that these pools contained high levels of seasonal influenza A RNA. In addition, RNase P was detected in all pools (median C_T_ = 19.9; IQR = 19.3 to 20.5), indicating adequate extraction and the absence of reaction inhibitors.

## DISCUSSION

This study demonstrates validation of pooled sample testing for influenza A(H5), which would support increased testing capacity for effective response in the event of an outbreak. Sample pooling necessarily involves dilution of sample material, leading to decreased analytical sensitivity that could potentially increase false-negative results (22). However, since the lower limit of detection of this assay remains at the low end of typical viral loads observed in clinical influenza A infections and historical human H5N1 cases (10^4^ to 10^8^ copies/mL), the clinical sensitivity should be sufficient (25, 26). While more complicated pooling schemes might provide additional efficiency gains, we used eight-sample pools to balance sensitivity, throughput, and operational simplicity (20). Although conjunctival swabs have been important for recent H5N1 cases given frequent conjunctival symptoms, our study focused on respiratory samples to support enhanced surveillance capacity in the event that H5N1 develops the ability to transmit between humans through infectious respiratory particles.

Limitations of this study include the use of a single inactivated virus strain that was culture-adapted and originally isolated from a bovine source. It will be important to evaluate the performance of this assay with eight-sample pools on true human clinical samples if an outbreak occurs. Such evaluation will help determine whether the pool size can be increased to expand capacity or should be reduced to improve sensitivity. In addition, quantitation of the virus stock relied on a ssDNA-based standard curve, which does not account for potential variability in RNA extraction or the efficiency of reverse transcription; however, this approach provides a consistent relative measure for determining the assay’s limit of detection for individual and pooled samples. Finally, given the constant evolution of influenza viruses, the primers and probes may require updates over time to maintain high performance.

H5N1 poses a potential pandemic threat, and testing capacity is currently limited. Pooled-sample testing is a practical strategy to increase testing throughput during disease outbreaks but requires proactive validation and implementation to be most effective. This pooled-sample approach for influenza A (H5) would increase testing capacity while maintaining adequate analytical sensitivity, enhancing our preparedness for potential outbreaks.

## Data Availability

All information necessary to evaluate the findings of this study is included in the article. Requests for additional information should be directed to the corresponding author.
